# PMAT2: An efficient graphical assembly toolkit for comprehensive organellar genomes

**DOI:** 10.1002/imt2.70064

**Published:** 2025-07-01

**Authors:** Fuchuan Han, Changwei Bi, Yicun Chen, Xiaogang Dai, Zefu Wang, Huaitong Wu, Ning Sun, Yanshu Qu, Yang Yang, Yangdong Wang, Tongming Yin

**Affiliations:** ^1^ State Key Laboratory of Tree Genetics and Breeding, Co‐Innovation Center for Sustainable Forestry in Southern China, Key Laboratory of Tree Genetics and Biotechnology of Educational Department of China, Key Laboratory of Tree Genetics and Silvicultural Sciences of Jiangsu Province Nanjing Forestry University Nanjing China; ^2^ Research Institute of Subtropical Forestry Chinese Academy of Forestry Hangzhou China; ^3^ College of Information Science and Technology & Artificial Intelligence Nanjing Forestry University Nanjing China; ^4^ Jiangxi Provincial Key Laboratory of Oil‐tea Camellia Resource Cultivation and Utilization Jiangxi Academy of Forestry Nanchang China

## Abstract

We developed PMAT2, an advanced toolkit for lineage‐specific de novo assembly of plant, animal, and fungal mitochondrial genomes, as well as plant chloroplast genome. PMAT2 leverages optimized graph‐based strategies tailored to organelle genome complexity, enabling complete and accurate assemblies, even with approximately 1 × highly accurate PacBio high‐fidelity (HiFi) reads. By assembling 150 organellar genomes across diverse lineages, PMAT2 outperformed existing tools in assembly completeness. The source code for PMAT2 is publicly available at https://github.com/aiPGAB/PMAT2.

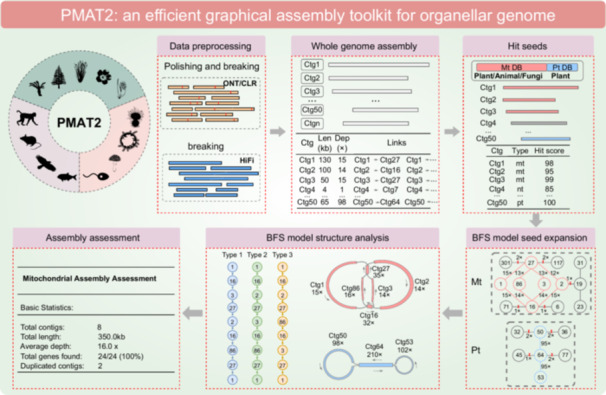

## AUTHOR CONTRIBUTIONS

**Fuchuan Han**: Writing—original draft; conceptualization; methodology; software; data curation; formal analysis; validation; investigation; visualization. **Changwei Bi**: Conceptualization; methodology; software; data curation; investigation; validation; formal analysis; writing—original draft; funding acquisition; writing—review and editing. **Yicun Chen**: Resources; project administration; funding acquisition. **Xiaogang Dai**: Validation; formal analysis. **Zefu Wang**: Validation; formal analysis. **Huaitong Wu**: Validation; formal analysis. **Ning Sun**: Validation; formal analysis. **Yanshu Qu**: Formal analysis; validation. **Yang Yang**: Validation; formal analysis. **Yangdong Wang**: Conceptualization; supervision; project administration; methodology; writing—review and editing. **Tongming Yin**: Supervision; project administration; methodology; conceptualization; writing—review and editing.

## CONFLICT OF INTEREST STATEMENT

The authors declare no conflicts of interest.

## ETHICS STATEMENT

No animals or humans were involved in this study.

To the Editor,

Mitochondria and chloroplasts, originating from endosymbiotic events involving an α‐proteobacterium and a cyanobacterium, respectively, are crucial for cellular energy production and photosynthesis [1, 2]. Organellar genomes, particularly mitochondrial genomes (mitogenomes), exhibit significant variation across eukaryotes [3]. Animal mitogenomes are generally compact, ranging from 11 to 50 kb, and typically encode 13 protein‐coding genes (PCGs), two ribosomal RNA (rRNA) genes, and 22 transfer RNA genes [4]. In contrast, fungal mitogenomes (12−213 kb) demonstrate considerable size variability, primarily due to the presence of introns and repetitive sequences [3]. Plant mitogenomes are characterized by their highly dynamic nature and expansive size range (66 kb−18 Mb) [5, 6]. This dynamism results in structural polymorphism, including circular, linear, or branched forms, driven by large noncoding repetitive regions [7, 8]. Additionally, plant mitogenomes also frequently harbor mitochondrial plastid DNAs (MTPTs) [9, 10, 11, 12]. Polyploidization and interspecific hybridization increase structural complexity by promoting genome doubling and recombination among repetitive sequences [13]. As a result, mitochondrial DNA from different ancestors may coexist in polyploid plants, forming complex reticulate genome structures.

Several bioinformatic tools, such as MitoHiFi and TIPPo, have been developed for mitogenome assembly [14, 15]. MitoHiFi specializes in analyzing assembly results from hifiasm and is particularly effective for assembling animal and fungal mitogenomes, which are generally small and often consist of a single contig. Its reference‐based approach enables accurate assembly in such cases. However, it is less suitable for plant mitogenomes, which are typically larger and structurally more complex. TIPPo is designed to analyze assembly results from Flye by classifying reads into nuclear, plastid, and mitochondrial origins before assembly. This method is highly dependent on the accuracy of read classification, which may be affected by the presence of MTPTs, nuclear plastid DNAs (NUPTs), nuclear mitochondrial DNAs (NUMTs), and rDNA clusters, and is thus prone to misclassification and potential structural omissions during assembly. PMAT1, a toolkit specifically developed for third‐generation sequencing data, addresses the challenges of plant mitogenome assembly and has been widely adopted in plant mitogenome research, significantly advancing this field [16]. However, the presence of abundant repetitive sequences and MTPTs in plant mitogenomes complicates the assembly process [8, 17]. When the depth difference between the organellar and nuclear genomes is minimal, PMAT1 struggles with seed sequence capture accuracy and processing efficiency, resulting in low assembly performance. Moreover, PMAT1 is not suitable for assembling animal and fungal mitogenomes, limiting its applicability across a broad range of species.

To overcome these limitations, we comprehensively enhanced PMAT1 and developed PMAT2, an advanced toolkit written in C. PMAT2 employs lineage‐specific assembly optimization strategies for plant, animal, and fungal mitogenomes, offering comprehensive optimization and enabling the high‐quality graphical assembly of organellar genomes, including specific optimization for plant chloroplast genome (plastomes). Using PMAT2, we successfully assembled 60 animal mitogenomes, 41 fungal mitogenomes, 25 plant mitogenomes, and 26 plant plastomes, achieving superior assembly completeness compared to other tools, often requiring only low (~1×) HiFi coverage for complete fungal and animal assemblies. This low‐depth assembly capability substantially reduces sequencing costs, facilitating large‐scale mitogenome and plastome assembly efforts, especially in studies involving numerous individuals or species under budget constraints.

## RESULTS AND DISCUSSION

### The PMAT2 workflow

**FIGURE 1:**
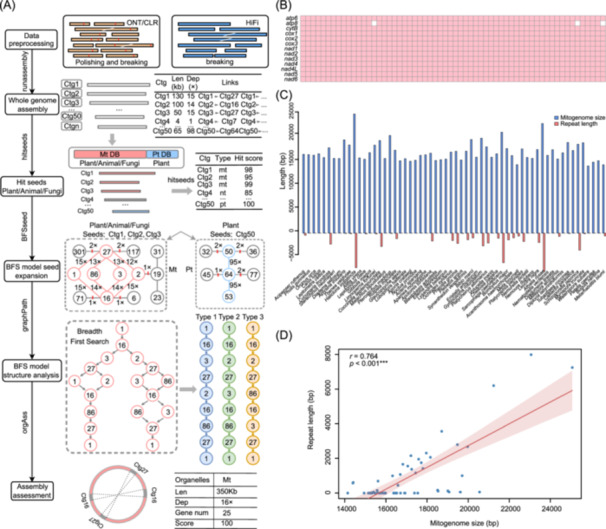
Assembly results of animal mitogenomes. (A) Workflow of PMAT2 toolkit. (B) The presence/absence of the 13 PCGs in 60 animal mitogenomes (with species in the same order as in panel C). (C) Blue indicates the total length of the mitogenome, and red indicates the total length of the repetitive sequences. (D) Fitted relationship between the total length of the mitogenomes and the total length of the repetitive sequences, with shaded areas indicating 95% confidence intervals (CIs). CLR, Continuous Long Reads; Ctg, Contig; Mt, Mitogenome; ONT, Oxford Nanopore Technologies; Pt, Plastome.

PMAT2 integrates optimized modules that address the key limitations of PMAT1, significantly improving mitogenome assembly accuracy and efficiency. Unlike TIPPo, which classifies reads before assembly, PMAT2 performs *de novo* assembly using all reads, avoiding the structural losses that can result from misclassification. PMAT2 reconstructs organelle genome paths by extending from high‐confidence seed contigs, overcoming the challenges MitoHiFi faces with complex organellar genomes. To efficiently handle graphical complexity, PMAT2 replaces PMAT1's exhaustive path‐search strategy with a breadth‐first search (BFS) algorithm, using hash tables for rapid path resolution. An automated evaluation process considers genome size, gene content, and contig‐mediated rearrangements, with conserved PCG detection serving as a proxy for completeness. MTPT‐induced misassemblies are minimized by tagging chloroplast‐derived sequences and adjusting contig scoring. PMAT2 supports two modes: autoMito for fully automated assembly and graphBuild for flexible, user‐guided reconstruction. Together, these advances enable accurate and efficient assembly across diverse mitogenome types, particularly in complex plant species. The PMAT2 pipeline consists of six tailored steps (Table S1 and Figure 1A).

### Step 1: Data preprocessing

For Oxford Nanopore Technologies and Continuous Long Reads, which are prone to higher error rates, PMAT2 first estimates the genome size by calculating the k‐mer frequency distribution. This estimation informs the subsequent self‐correction of reads, which is performed using either NextDenovo or Canu to enhance sequence accuracy by correcting sequencing errors [18, 19]. For HiFi reads, which inherently exhibit higher accuracy, the correction step is skipped, and the pipeline proceeds directly to the assembly phase. To handle large datasets efficiently and reduce computational burden, PMAT2 allows users to subsample the input data using a subsampling parameter, ensuring efficient processing while maintaining sufficient coverage for accurate assembly.

### Step 2: Whole‐genome assembly

Following preprocessing, PMAT2 performs whole‐genome *de novo* assembly to generate initial contigs. The corrected reads (for Oxford Nanopore Technologies and Continuous Long Reads) or raw HiFi reads are assembled into contigs, which serve as the foundation for subsequent seed sequence identification and mitogenome reconstruction. The *de novo* assembly module used here is identical to that implemented in PMAT1. This step produces a set of contigs that are further processed in the downstream steps of the pipeline.

### Step 3: Find candidate contigs

In this phase, PMAT2 employs a sophisticated scoring module to select candidate contigs for mitogenome assembly. A composite score is calculated for each contig by integrating three key parameters: sequencing depth, contig length, and mitochondrial gene content. Each parameter is normalized and processed through a sigmoid function to ensure consistent scaling across contigs with diverse characteristics. To accommodate lineage‐specific mitogenome properties, the relative weights of these parameters are dynamically adjusted based on the user‐specified taxonomic group. For plant mitogenomes, where MTPTs are prevalent, contigs harboring chloroplast‐derived sequences are identified through gene annotation and assigned reduced weights in the scoring process. To mitigate interference from large non‐mitochondrial contigs containing homologous sequences, gene content is prioritized over contig length in the scoring algorithm. Contigs are ranked according to their composite scores, and those surpassing predefined thresholds are selected as seeds for subsequent mitogenome reconstruction.

### Step 4: BFS model seed expansion

In the seed expansion phase, PMAT2 employs a BFS algorithm, implemented via the BFSseed module, to extend mitogenome paths from high‐confidence seed contigs. This represents a significant improvement over PMAT1, which relied on the sequential traversal of all nodes with a query time complexity of O(n). By leveraging efficient hash‐based data structures, the BFSseed module enables rapid access to node connectivity information, thereby markedly enhancing the efficiency of path discovery in complex and repetitive genomic regions.

During path extension, the BFSseed module evaluates both contig depth and path (link) depth to ensure the biological validity of each extension. For plastome assembly, contigs with a sequencing depth below 0.3 times the depth of the best‐matching seed contig are excluded from further analysis. For mitogenome assembly, contigs exceeding twice the estimated average genome depth are deemed unreliable and filtered out. This dual‐threshold strategy minimizes the inclusion of artifacts and contaminants, thereby improving assembly accuracy.

### Step 5: BFS model structure analysis

To simplify and resolve the assembly graph, PMAT2 introduces the bfsMap module, a hash‐table‐based graph path resolution method designed for complex and multi‐circular structures. The algorithm starts by calculating the maximum repeat count for each sequence in the assembly graph, then selects the longest sequence as the starting point and dynamically expands to adjacent nodes using a hash table, with path information stored in bfsPath. Path priority is determined based on a combination of path length, node count, and path depth, allowing the algorithm to evaluate multiple candidate paths and identify the optimal path. This process enables bfsMap to effectively resolve repeat‐induced cycles and accurately output the complete structure of organelle genomes. The resulting path reflects a circular or multi‐circular genome configuration, providing a more precise and interpretable representation of the true mitochondrial or plastid genome structure.

### Step 6: Assembly assessment

The final step of the PMAT2 pipeline uses the orgAss module to assess and output the assembled organellar genomes. It retrieves annotation data and evaluates key metrics such as gene content, mitogenome depth, assembly length, number of contigs involved in rearrangements, and path depth to determine assembly quality. The resulting sequence is provided in a standard format along with a detailed quality report, supporting further optimization and ensuring suitability for downstream analyses such as phylogenetics or functional genomics.

### PMAT2 performance in organellar genome assembly

To evaluate the accuracy of PMAT2 in assembling animal mitogenomes, we successfully assembled mitogenomes from 60 species across nine phyla (Table S2). The assembled mitogenomes ranged in size from 14,144 to 25,064 bp. The assembly results showed high consistency with published mitogenome lengths, except for three species lacking published data. Only one species exhibited a linear structure; the remaining 59 possessed a single circular structure, consistent with typical animal mitogenome architecture (Figure S1). Annotation analysis revealed successful capture of the 13 core PCGs in most species; only *Limnoperna fortunei*, *Schizoporella japonica*, and *Metarhabditis blumi* lacked the *atp8* gene, indicating high annotation completeness (Figure 1B). *Athalia rosae* contained the longest repetitive sequence (7982 bp; Table S3 and Figure 1C). Analysis of repetitive sequences (>30 bp) revealed a strong positive correlation between total repeat length and mitogenome size (*r* = 0.764, *p* < 2e‐16; Figure 1D). Assembly contiguity was high, with only seven species requiring multiple contigs.

For 41 fungal species (18 Ascomycota, 18 Basidiomycota, 4 Zygomycota, 1 Blastocladiomycota; 18 newly assembled), mitogenomes showed substantial length variation, from 24,299 bp (*Trichophyton mentagrophytes*) to 156,349 bp (*Ceratobasidium cereale*) (Table S4). PMAT2 assembly results were highly consistent with published lengths. Annotation analysis showed most species had all PCGs identified (Figure S2A), accurately reflecting known variations like the lack of *nad1‐5* and *nad4L* in *Lachancea thermotolerans*. *Ceratobasidium cereale* contained 82,815 bp of repetitive sequences (Table S5), accounting for 53% of its mitogenome (Figure S2B). Similar to animals, a significant correlation existed between total repeat length and fungal mitogenome length (*r* = 0.731, *p* < 2e‐16; Figure S2C). Only two species exhibited a linear structure; the rest formed a single circular structure (Figure S2D). Fungal assembly often required fewer reads than animals.

In the analysis of 26 plant species (4 Algae, 11 Bryophytes, 2 Lycophytes, 1 Monilophyta, 3 Gymnospermae, 5 Angiospermae; 13 plastomes and 11 mitogenomes newly assembled), PMAT2 and TIPPo exhibited similar accuracy in plastome assembly, consistently generating circular structures consistent with references (Figure S3 and Table S6). Plastomes showed a moderate positive correlation between repeat length and total length (*r* = 0.609, *p* = 0.00159; Figure S4A). Most plastomes exhibited the typical quadripartite structure (one large single copy, one small single copy, and two inverted repeats), except for two Algae and one Gymnosperm (Figure S4B). Compared to TIPPo, PMAT2 showed higher assembly success, completing 25 of 26 mitogenomes, while TIPPo completed only 14. Both tools failed to assemble the mitogenome of *Cupressus sempervirens*, likely due to the low coverage (<0.6×). Mitogenome repeat length showed a strong positive correlation with total length (*r* = 0.875, *p* < 2e‐16; Figure S4C). Mitogenome structures varied: Bryophyte and Algae typically assembled into a single circle, while Angiospermae, Lycophytes, and Monilophyta displayed more complex configurations (Figure S4D). MTPT analysis across 21 species showed higher abundance in three Angiosperms (*Amaranthus tricolor*, *Malus domestica*, and *Juncus effusus*) and *Asplenium marinum* (Monilophyta), with *A. marinum* containing the longest MTPTs (52,645 bp, 34% of its plastome length; Table S7). Algae, Bryophytes, and Gymnospermae displayed fewer and shorter MTPTs.

### PMAT2 performance with varying read depths and multicircular mitogenome structures

**FIGURE 2:**
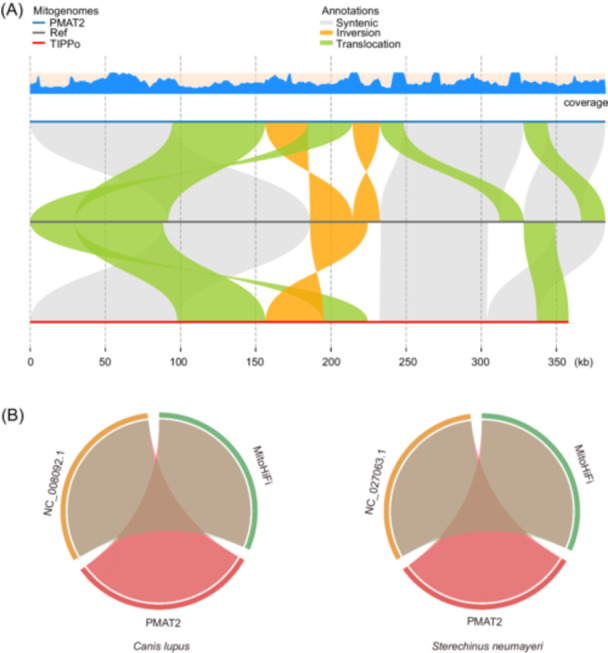
Assembly and collinearity results of the mitogenome. (A) Collinearity results of the *Amaranthus tricolor* mitogenome; the blue coverage curve indicates the sequencing depth of the PMAT2 assembly sequence. (B) Collinearity results for mitogenomes under low sequencing depth.

To further assess the capability of PMAT2 in resolving multicircular mitogenome structures, its performance was compared with TIPPo using *Jasminum sambac* and *Amaranthus tricolor*. For *J. sambac*, both tools successfully assembled two circular structures consistent with previous studies (Figure S5) [20]. However, notable differences were observed in *A. tricolor*, where PMAT2 accurately generated four circular structures totaling 382,390 bp, perfectly matching the reference (Figure S5). In contrast, TIPPo produced three circular and one linear structure totaling 385,042 bp, omitting two segments of 23,258 and 47,094 bp (Figure 2A). This discrepancy is likely due to the reliance of TIPPo on pre‐assembly read classification and filtering, a strategy that is highly sensitive to classification accuracy. In contrast, PMAT2 adopts a graph‐based assembly approach that preserves all contig connections. This allows the reconstruction of complete mitogenomes by resolving genome paths from the graph. The graphical representation helps retain complex structures, especially those involving repeat‐mediated rearrangements, and avoids the loss of alternative configurations.

PMAT2 demonstrated robust performance under low sequencing depth. Minimum‐depth tests using random sampling on 16 species compared PMAT2 with MitoHiFi. Both produced assemblies highly consistent with references for the species they successfully assembled (Figures 2B and S6, Table S8). However, MitoHiFi failed to assemble four species (*Diadema setosum*, *L. fortunei*, *Nemurella pictetii*, and *Pleurotus ostreatus*) where PMAT2 succeeded (Table S8). Notably, for *P. ostreatus*, under the lowest tested sequencing depth (2 Mb, ~0.08×), PMAT2 successfully generated a complete assembly. MitoHiFi demonstrated significantly faster runtimes on larger datasets due to its reference‐based filtering strategy. In this approach, HiFi reads are aligned to a closely related mitogenome, and reads exceeding the reference length are removed before assembly. This reduces input data and shortens computation time. However, the method relies on the availability of suitable references and assumes high sequence similarity. In species without a close reference or with substantial structural variation, informative reads may be mistakenly discarded, resulting in incomplete or failed assemblies. In contrast, PMAT2 performs full *de novo* assembly using all available reads, ensuring no potentially informative sequences are excluded. Although this leads to longer runtimes as data volume increases, it allows successful assembly across a wider range of species, especially those lacking appropriate references. Runtime can be reduced by subsampling reads, enabling PMAT2 to match MitoHiFi in speed. For instance, at the lowest sequencing depth tested for *P. ostreatus*, PMAT2 completed the assembly in just 2 s. Moreover, mitogenomes often evolve rapidly in structure but remain conserved at the sequence level. PMAT2 takes advantage of this by identifying conserved protein‐coding sequences as seeds and reconstructing the genome through a breadth‐first graph traversal algorithm. This approach enables accurate recovery of complete mitochondrial structures, even in the presence of complex rearrangements.

Collinearity analyses revealed that, except for a discrepancy in *Tricholoma terreum* (where PMAT2 demonstrated higher consistency with the reference), assemblies generated by both tools were generally well‐aligned with reference genomes for the 12 species examined (Figures 2B and S6). These results highlight the accuracy, completeness, and broad applicability of PMAT2, particularly under low‐depth sequencing conditions and in challenging assembly scenarios.

## CONCLUSION

PMAT2 is a graphical organelle genome assembly tool specifically designed for the efficient assembly of mitogenomes from plants, animals, and fungi, as well as plant plastomes. It incorporates advanced functionalities to resolve complex mitogenome structures and evaluate mitogenome completeness. PMAT2 outperforms existing assembly tools in terms of both the accuracy and completeness of its assembly results. In summary, PMAT2 provides a robust and efficient solution for organelle genome assembly, addressing key challenges in the field.

## METHODS

Detailed procedures for sample/data collection, sequencing protocol, and bioinformatic and statistical analysis approaches are available in the Supporting Information.

## Supporting information


**Figure S1.** The assembly graph of animal mitogenome.
**Figure S2.** Assembly results of 41 fungal mitogenomes.
**Figure S3.** Assembly graph of plant organelle genomes constructed by TIPPo, with the chloroplast genome depicted above the dashed line and the mitochondrial genome below.
**Figure S4.** Assembly results of 26 plant organellar genomes.
**Figure S5.** Assembly graphs of organelle genomes for *Amaranthus tricolor* and *Jasminum sambac*, generated by PMAT2 and TIPPo, respectively.
**Figure S6.** Collinearity results for the mitogenomes of 12 species.

**Table S1.** Comparison of PMAT1 and PMAT2 functional modules.
**Table S2.** Sequencing and assembly statistics of 60 animal mitochondrial genome.
**Table S3.** Repeat sequence statistics of 60 animal mitochondrial genomes.
**Table S4.** Sequencing and assembly statistics of 41 fungal mitochondrial genome.
**Table S5.** Repeat sequence statistics of 41 fungal mitochondrial genomes.
**Table S6.** Sequencing and assembly statistics of 26 plant organellar genomes.
**Table S7.** Length statistics of MTPTs in 21 plant mitochondrial genomes.
**Table S8.** The results of the 16 species run at the lowest sequencing depths.

## Data Availability

The data that supports the findings of this study are available in the supplementary material of this article. The HiFi datasets for animals and fungi used in this study were obtained from the NCBI database. Specific download IDs can be found in Tables S2, S4, and S6. The scripts of PMAT2 are available at https://github.com/aiPGAB/PMAT2. Supplementary materials (methods, figures, tables, graphical abstract, slides, videos, Chinese translated version, and update materials) may be found in the online DOI or iMeta Science http://www.imeta.science/.
