# Supplementary material for: PMAT2: An efficient graphical assembly toolkit for comprehensive organellar genomes
Source: Imeta. 2025 Jul 1;4(4):e70064. doi: 10.1002/imt2.70064 (PMC12371249; doi:10.1002/imt2.70064)
Supplement: Supplementary file 1 — Figure S1. The assembly graph of animal mitogenome. Figure S2. Assembly results of 41 fungal mitogenomes. Figure S3. Assembly graph of plant organelle genomes constructed by TIPPo, with the chloroplast genome depicted above the dashed line and the mitochondrial genome below. Figure S4. Assembly results of 26 plant organellar genomes. Figure S5. Assembly graphs of organelle genomes for Amaranthus tricolor and Jasminum sambac, generated by PMAT2 and TIPPo, respectively. Figure S6. Collinearity results for the mitogenomes of 12 species. [file IMT2-4-e70064-s002.docx]

Supporting Information to

**PMAT2: an efficient graphical assembly toolkit for comprehensive organellar genomes**

**Running title**: comprehensive organellar genome assembly toolkit

Fuchuan Han^1,2#^, Changwei Bi^1,3#*^, Yicun Chen^2^, Xiaogang Dai^1^, Zefu Wang^1^, Huaitong Wu^1^, Ning Sun^3^, Yanshu Qu^1,4^, Yang Yang^2^, Yangdong Wang^2*^, Tongming Yin^1*^

^1^State Key Laboratory of Tree Genetics and Breeding, Co-Innovation Center for Sustainable Forestry in Southern China, Key Laboratory of Tree Genetics and Biotechnology of Educational Department of China, Key Laboratory of Tree Genetics and Silvicultural Sciences of Jiangsu Province, Nanjing Forestry University, Nanjing 210037, China

^2^Research Institute of Subtropical Forestry, Chinese Academy of Forestry, Hangzhou 311400, China

^3^College of Information Science and Technology & Artificial Intelligence, Nanjing Forestry University, Nanjing 210037, China

^4^Jiangxi Provincial Key Laboratory of Oil-tea Camellia Resource Cultivation and Utilization, Jiangxi Academy of Forestry, Nanchang 330032, China

^#^These authors contributed equally: Fuchuan Han; Changwei Bi

*Correspondence: tmyin@njfu.com.cn (Tongming Yin); wangyangdong@caf.ac.cn (Yangdong Wang); bichwei@njfu.edu.cn (Changwei Bi)

**METHODS**

**Organelle genome assembly and repetitive sequence identification**

The HiFi datasets for animals and fungi used in this study were obtained from the NCBI database. Specific download IDs can be found in Tables S2, S4, and S6. Organelle genomes of animal species were assembled using the autoMito mode of the PMAT2 software with parameters “autoMito -x 1 -m”, while fungal genomes were assembled using “autoMito -x 2 -m”, both combined with the “-F” parameter for dataset subsampling. Annotation of animal and fungal genomes was performed using the local version of the MITOS2 software [1]. For plant species, organelle genomes were assembled using both PMAT2 and TIPPo, with PMAT2 parameters set as “autoMito -x 0 -m -t 10” and TIPPo parameters set as “-t 10” [2]. The sequencing data were aligned back to the assembled mitogenome using minimap2, and the coverage depth was calculated using samtools with a sliding window size of 1000 bp [3,4]. The assembled results were compared for collinearity using Mummer4 and visualized with plotsr [5]. Assembly graphs for all organelle genomes were visualized using Bandage [6].

To identify repetitive sequences, BlastN v2.14.0 was used to search for sequences longer than 30 bp. The search parameters were set as “-word_size 7 -evalue 1e-6” [7]. The matching repetitive sequences were further sorted and merged to avoid duplicate calculations of sequences in the same position.

To assess the relationship between repetitive sequence length and mitogenome size, a least squares linear regression model was applied, and Pearson correlation coefficients were calculated. *p*-values from correlation analyses were adjusted using the Benjamini–Hochberg method to control the false discovery rate (FDR). Adjusted *p*-values were reported, and significance was determined at an FDR threshold of 0.05. The 95% confidence interval (CI) for the regression was also calculated.

**Identification of MTPTs**

To identify MTPTs in plant mitogenomes, BlastN v2.14.0 was used with plastome sequences as the query and mitogenome sequences as the subject [7]. MTPTs were filtered based on the following criteria: fragment length ≥ 50 bp, sequence identity ≥ 80%, and E-value ≤ 1e-5. The matching fragments were further sorted by their start positions in the plastome and merged to avoid redundant calculations of overlapping regions.

**Impact of read depth on organelle assembly**

To assess the impact of read depth on PMAT2 assembly accuracy, the random sampling function of PMAT2 was used to subsample the datasets. The specific sampling depths and reference genome information are shown in Table S8. The subsampled data were used for assembly with both PMAT2 and MitoHiFi v3.2.2 [8]. For animal species, the PMAT2 parameters were “autoMito -x 2 -T 10 -m”, while MitoHiFi was set to “-r ref.fasta -g ref.gb -t 10 -a animal”. For fungal species, the PMAT2 parameters were “autoMito -x 1 -T 10 -m”, and MitoHiFi parameters were “-r ref.fasta -g ref.gb -t 10 -a fungi”. Assembly results were compared using the Mummer4 tool to evaluate assembly consistency [9]. The parameters used were set as “-i 90 -l 100”. The comparison results were then visualized using Circos v0.69.9 [10].

**REFERENCES**

1. Bernt, Matthias, Alexander Donath, Frank Jühling, Fabian Externbrink, Catherine Florentz, Guido Fritzsch, Joern Pütz, Martin Middendorf, Peter F. Stadler. 2013. “MITOS: improved de novo metazoan mitochondrial genome annotation.” *Molecular Phylogenetics and Evolution* 69: 313-319. https://doi.org/10.1016/j.ympev.2012.08.023

2. Xian, Wenfei, Ilja Bezrukov, Zhigui Bao, Sebastian Vorbrugg, Anupam Gautam, Detlef Weigel. 2025. “TIPPo: a user-friendly tool for de novo assembly of organellar genomes with high-fidelity data.” *Molecular Biology and Evolution* 42: msae247. https://doi.org/10.1093/molbev/msae247

3. Li, Heng. 2018. “Minimap2: pairwise alignment for nucleotide sequences.” *Bioinformatics* 34: 3094-3100. https://doi.org/10.1093/bioinformatics/bty191

4. Danecek, Petr, James K. Bonfield, Jennifer Liddle, John Marshall, Valeriu Ohan, Martin O. Pollard, Andrew Whitwham, et al. 2021. “Twelve years of SAMtools and BCFtools.” *GigaScience* 10: giab008. https://doi.org/10.1093/gigascience/giab008

5. Goel, Manish, Korbinian Schneeberger. 2022. “plotsr: visualizing structural similarities and rearrangements between multiple genomes.” *Bioinformatics* 38: 2922-2926. https://doi.org/10.1093/bioinformatics/btac196

6. Wick, Ryan R., Mark B. Schultz, Justin Zobel, Kathryn E. Holt. 2015. “Bandage: interactive visualization of *de novo* genome assemblies.” *Bioinformatics* 31: 3350-3352. https://doi.org/10.1093/bioinformatics/btv383

7. Ye, Jian, Scott McGinnis, Thomas L. Madden. 2006. “BLAST: improvements for better sequence analysis.” *Nucleic Acids Research* 34: W6-W9. https://doi.org/10.1093/nar/gkl164

8. Uliano-Silva, Marcela, João Gabriel R. N. Ferreira, Ksenia Krasheninnikova, Darwin Tree of Life Consortium, Mark Blaxter, Nova Mieszkowska, Neil Hall, et al. 2023. “MitoHiFi: a python pipeline for mitochondrial genome assembly from PacBio high fidelity reads.” *BMC Bioinformatics* 24: 288. https://doi.org/10.1186/s12859-023-05385-y

9. Marçais, Guillaume, Arthur L. Delcher, Adam M. Phillippy, Rachel Coston, Steven L. Salzberg, Aleksey Zimin. 2018. “MUMmer4: a fast and versatile genome alignment system.” *PLOS Computational Biology* 14: e1005944. https://doi.org/10.1371/journal.pcbi.1005944

10. Krzywinski, Martin, Jacqueline Schein, Inanç Birol, Joseph Connors, Randy Gascoyne, Doug Horsman, Steven J. Jones, Marco A. Marra. 2009. “Circos: an information aesthetic for comparative genomics.” *Genome Research* 19: 1639-1645. https://doi.org/10.1101/gr.092759.109


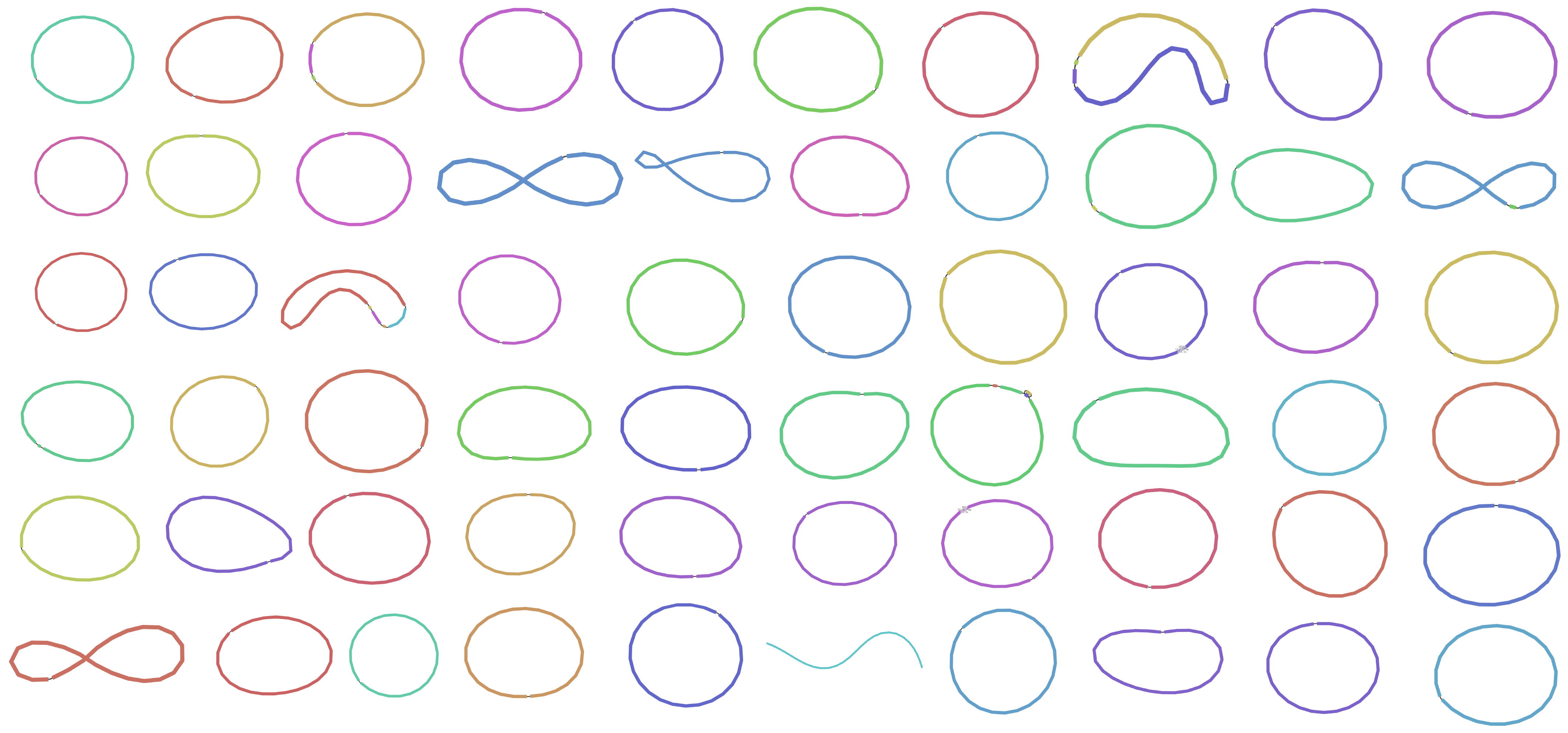


**Figure S1** The assembly graph of animal mitogenome.


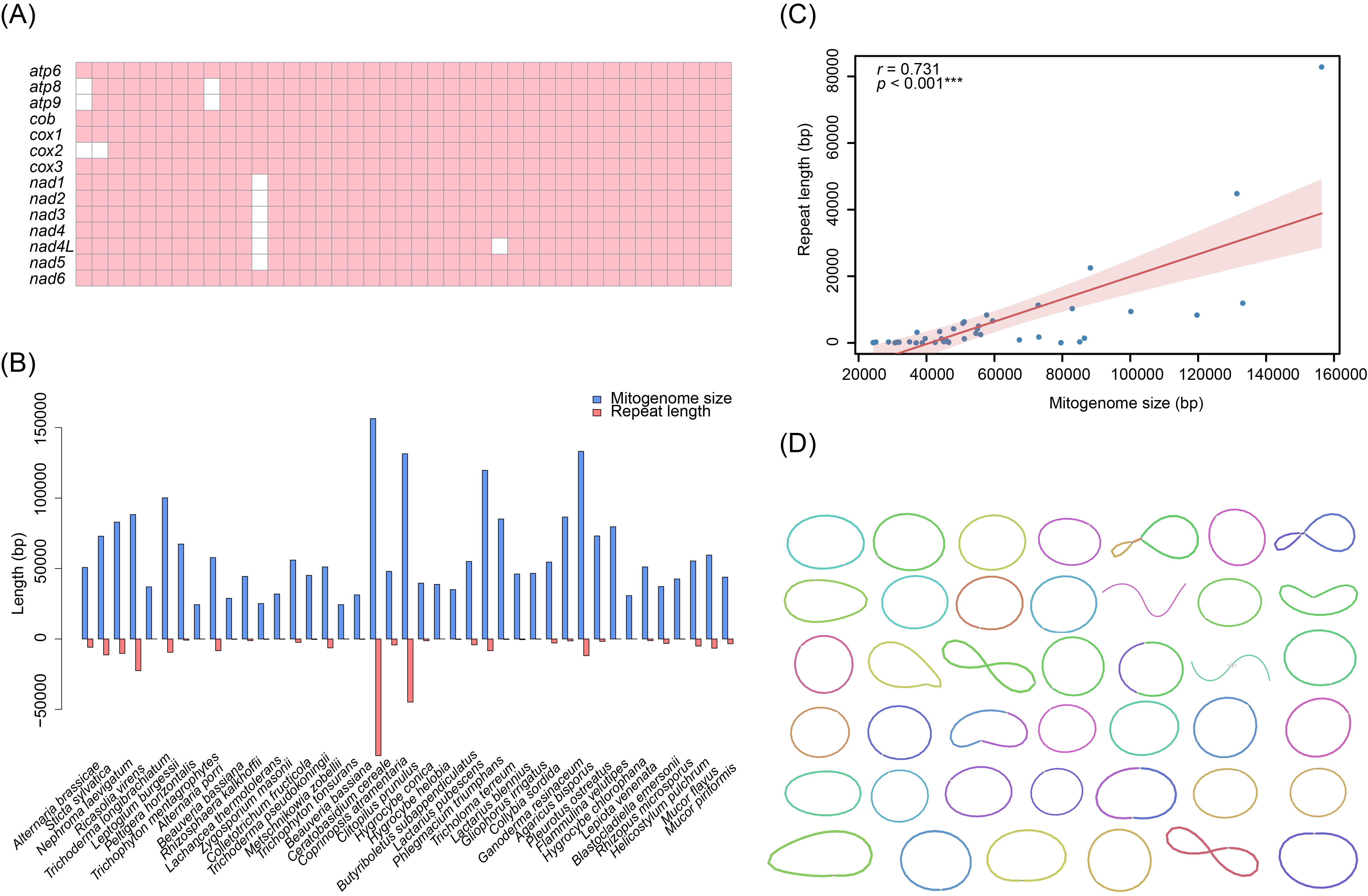


**Figure S2** Assembly results of 41 fungal mitogenomes. (A) The distribution of the content of the 14 PCGs in the 41 fungal mitogenomes (with species in the same order as in panel B). (B) The total length of the mitogenome is indicated in blue, and the total length of the repetitive sequences is indicated in red. (C) The fitted relationship between the total length of the mitogenome and the total length of the repetitive sequences, with shaded areas indicating 95% CIs. (D) The assembly graph of the mitogenome.


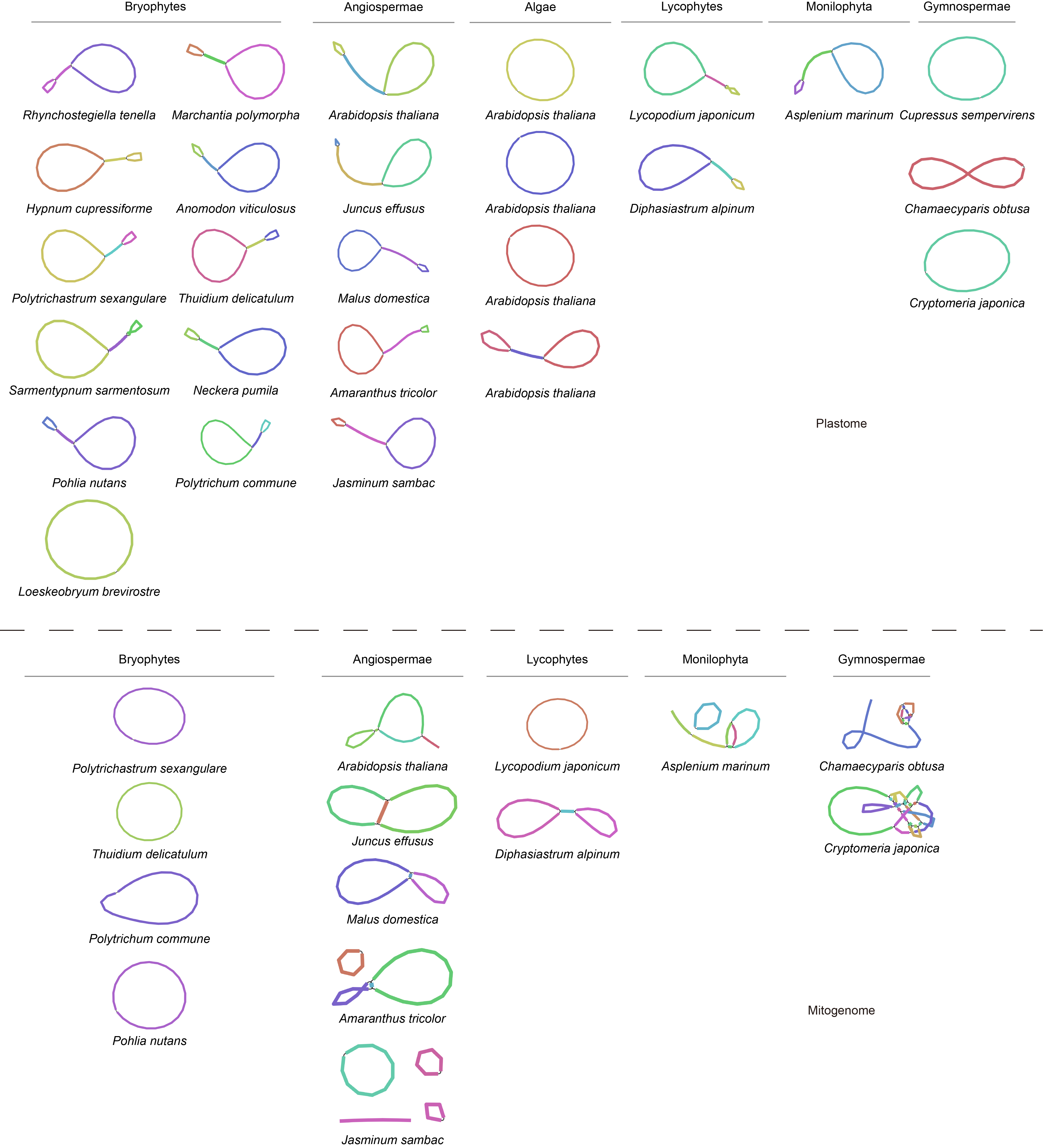


**Figure S3** Assembly graph of plant organelle genomes constructed by TIPPo, with the chloroplast genome depicted above the dashed line and the mitochondrial genome below.


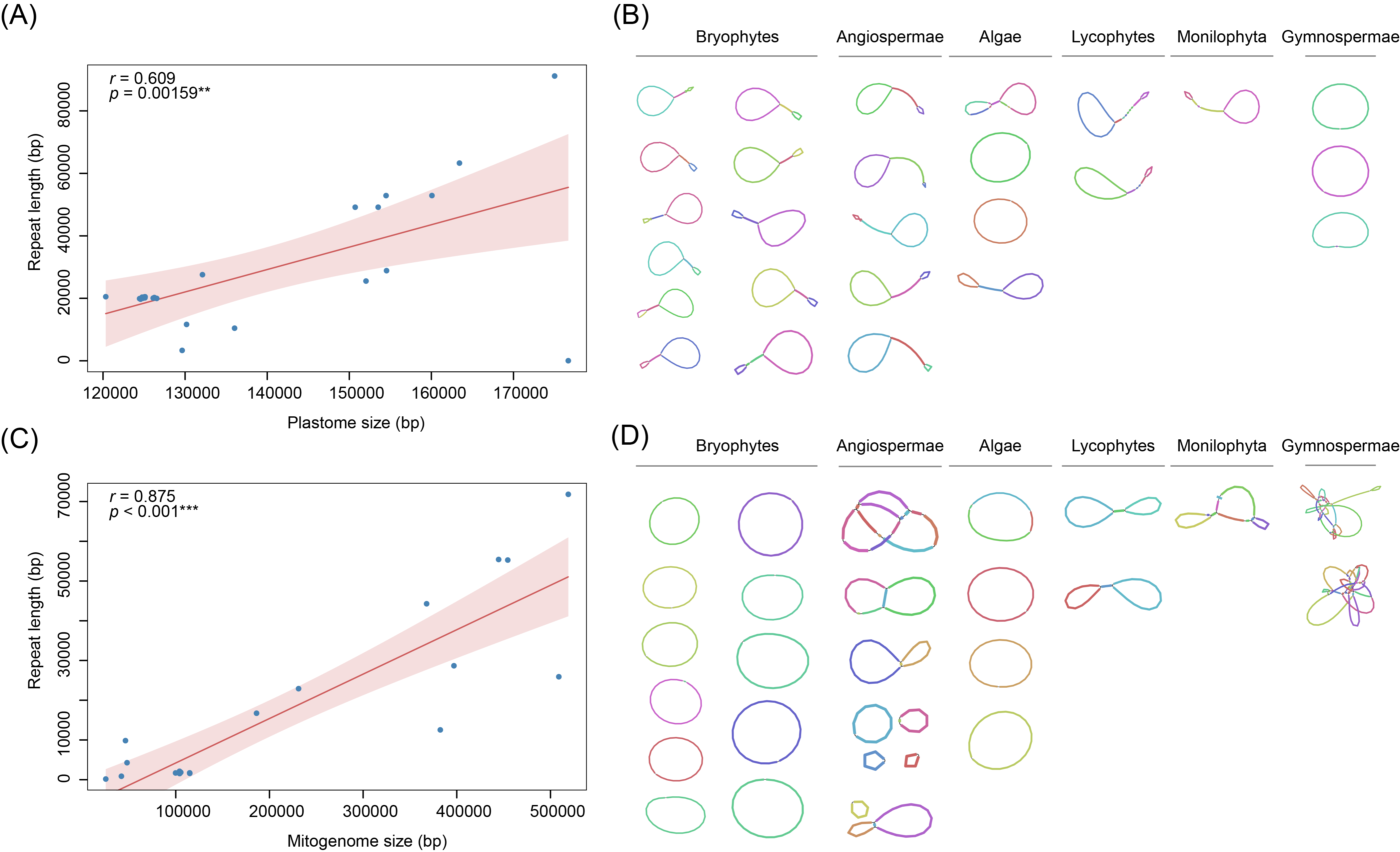


**Figure S4** Assembly results of 26 plant organellar genomes. (A) The fitted relationship between the total length of the plastomes and the total length of the repetitive sequences, with shaded areas indicating 95% CIs. (B) The assembly graph of the plastomes generated by PMAT2. (C) The fitted relationship between the total length of the mitogenomes and the total length of the repetitive sequences, with shaded areas indicating 95% CIs. (D) The assembly graph of the mitogenomes generated by PMAT2.


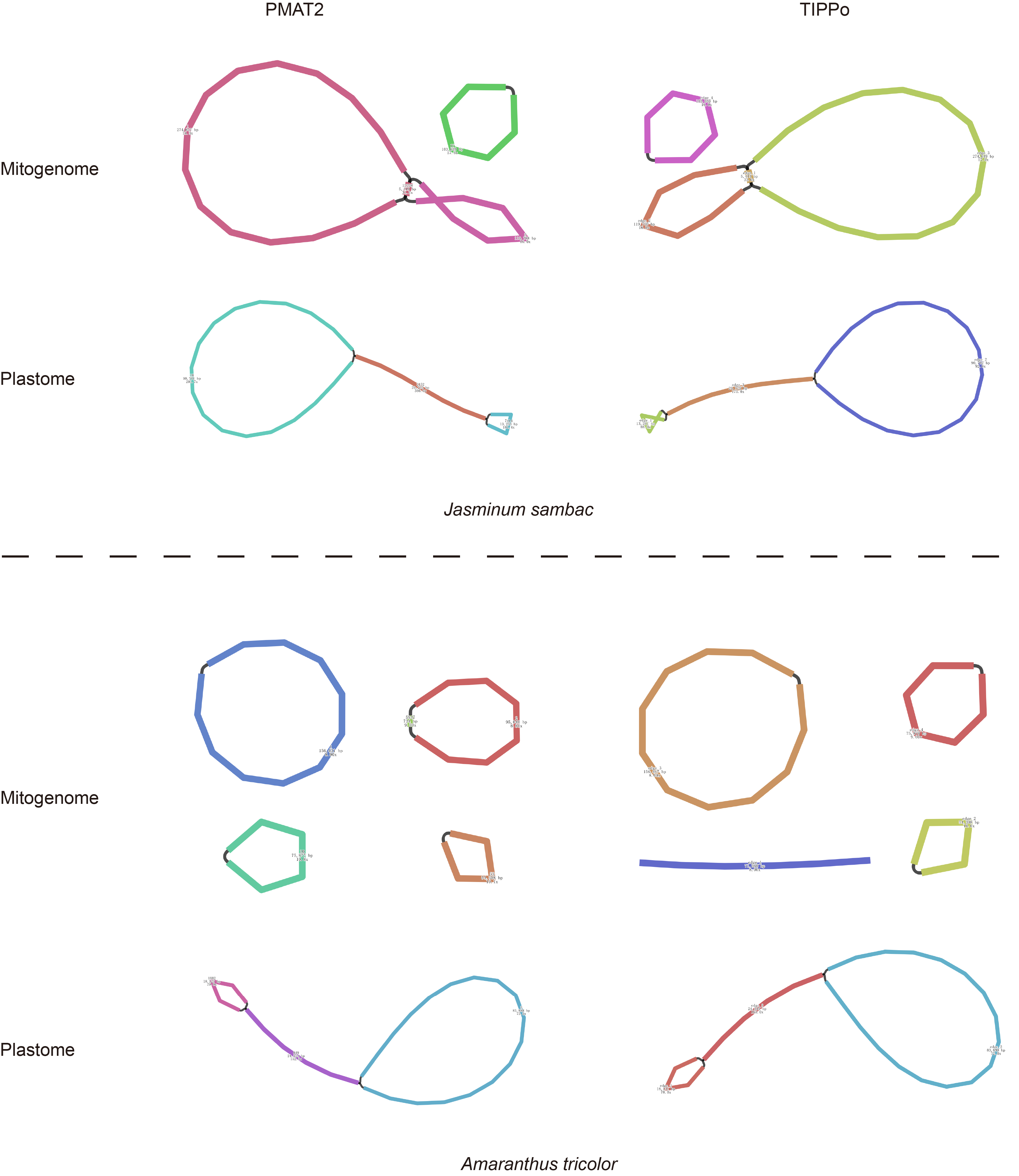


**Figure S5** Assembly graphs of organelle genomes for *Amaranthus tricolor* and *Jasminum sambac*, generated by PMAT2 and TIPPo, respectively.


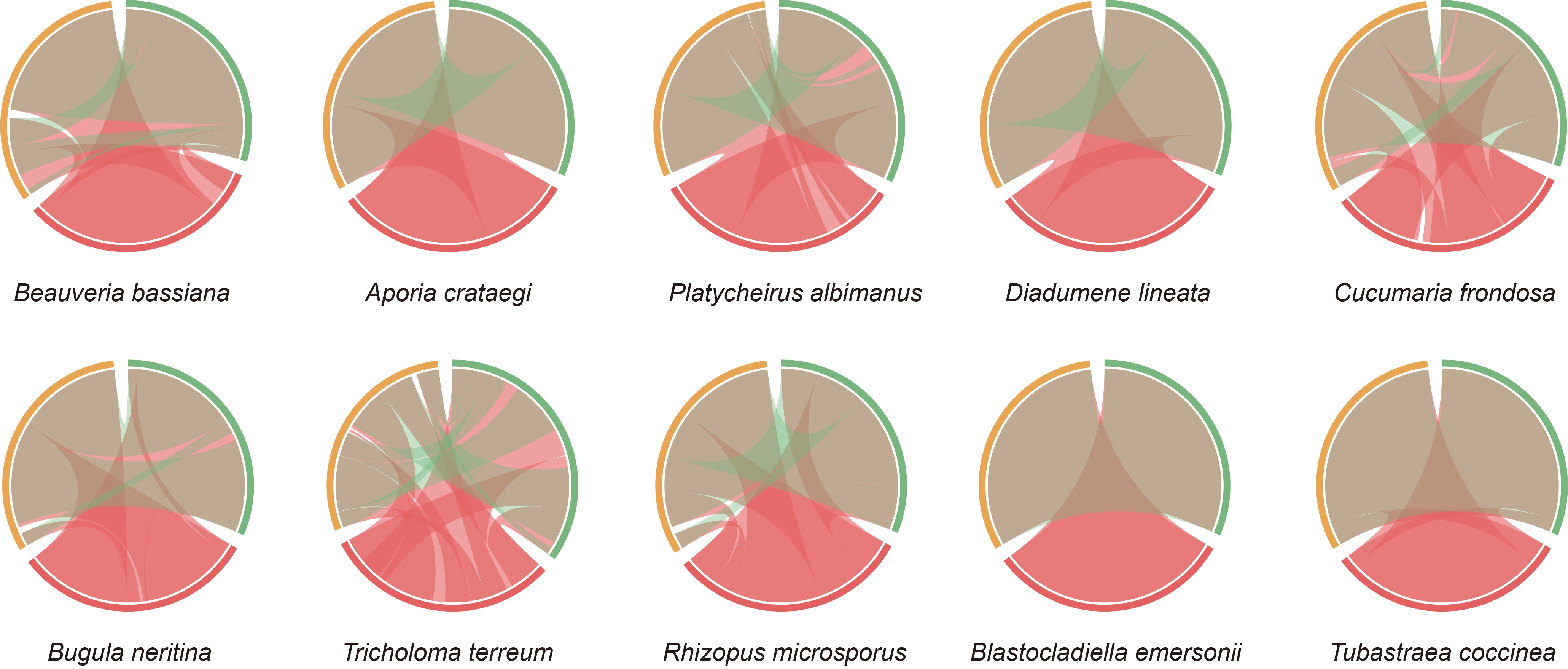


**Figure S6** Collinearity results for the mitogenomes of 12 species. Red represents PMAT2, green represents MitoHiFi, and orange represents the reference sequence.
